# The Composites of Polyamide 12 and Metal Oxides with High Antimicrobial Activity

**DOI:** 10.3390/polym14153025

**Published:** 2022-07-26

**Authors:** Paulina Latko-Durałek, Michał Misiak, Monika Staniszewska, Karina Rosłoniec, Marta Grodzik, Robert P. Socha, Marcel Krzan, Barbara Bażanów, Aleksandra Pogorzelska, Anna Boczkowska

**Affiliations:** 1 Faculty of Materials Science and Engineering, Warsaw University of Technology, Wołoska 141 Street, 02-507 Warsaw, Poland; michal.misiak.dokt@pw.edu.pl (M.M.); anna.boczkowska@pw.edu.pl (A.B.); 2 Centre for Advanced Materials and Technologies CEZAMAT, Poleczki 19 Street, 02-822 Warsaw, Poland; monika.staniszewska@pw.edu.pl (M.S.); karina.rosloniec@pw.edu.pl (K.R.); 3 Institute of Biology, Warsaw University of Life Sciences, Nowoursynowska 166 Street, 02-787 Warsaw, Poland; marta_grodzik@sggw.edu.pl; 4 Research and Development Center of Technology for Industry, Ludwika Waryńskiego 3A Street, 00-645 Warsaw, Poland; robert.socha@cbrtp.pl; 5 Jerzy Haber Institute of Catalysis and Surface Chemistry, Polish Academy of Sciences, Niezapominajek 8 Street, 30-239 Kraków, Poland; marcel.krzan@ikifp.edu.pl; 6 Faculty of Veterinary Medicine, Division of Microbiology, Wrocław University of Environmental and Life Sciences, C.K. Norwida 31 Street, 50-452 Wrocław, Poland; barbara.bazanow@upwr.edu.pl (B.B.); aleksandra.pogorzelska@upwr.edu.pl (A.P.)

**Keywords:** thermoplastic composites, polyamide 12, metal oxides, pathogens, toxicity, rheology, thermal properties, microstructure

## Abstract

The lack of resistance of plastic objects to various pathogens and their increasing activity in our daily life have made researchers develop polymeric materials with biocidal properties. Hence, this paper describes the thermoplastic composites of Polyamide 12 mixed with 1–5 wt % of the nanoparticles of zinc, copper, and titanium oxides prepared by a twin-screw extrusion process and injection moulding. A satisfactory biocidal activity of polyamide 12 nanocomposites was obtained thanks to homogenously dispersed metal oxides in the polymer matrix and the wettability of the metal oxides by PA12. At 4 wt % of the metal oxides, the contact angles were the lowest and it resulted in obtaining the highest reduction rate of the *Escherichia coli* (87%), *Candida albicans* (53%), and *Herpes simplex 1* (90%). The interactions of the nanocomposites with the fibroblasts show early apoptosis (11.85–27.79%), late apoptosis (0.81–5.04%), and necrosis (0.18–0.31%), which confirms the lack of toxicity of used metal oxides. Moreover, the used oxides affect slightly the thermal and rheological properties of PA12, which was determined by oscillatory rheology, thermogravimetric analysis, and differential scanning calorimetry.

## 1. Introduction

Polymer and polymer-based composites have gained tremendous popularity as materials for the production of everyday appliances such as computers, pens, cases, packaging, toys, etc., due to their low price, good mechanical properties, high corrosion resistance, and easy shaping. However, one of their main drawbacks is the lack of any resistance to microorganisms. With their popularity in our everyday lives and presence in public spaces, pathogens such as fungi, viruses, and bacteria can settle on their surfaces and be spread rapidly by people. Obviously, the pathogens transmission will be dependent on the survivability of the pathogens on the surface. The longer they live, the higher risk that pathogens will be transmitted. It was examined that the virus of SARS-CoV-2 remained much longer (8 h) on a plastic surface compared to the other surfaces such as aluminum, copper, steel, rubber, cloth materials, and paper [1]. Moreover, a whole range of factors such as humidity, temperature, dust, sweat, dead skin cells or food particles contribute to rapid multiplication of pathogens. It is known that pathogens are mainly present in hospitals, and are responsible for numerous infections because they remain on plastic objects such as bedside rails, tables, floors. Besides, thermoplastic polymers are used to produce various types of small medical devices such as syringes, catheters, urine collectors, volumetric infusion pumps, packages as well as personal protective equipment including masks, gloves, and disposable aprons [2,3]. For instance, the Methicillin-Resistant *Staphylococcus aureus* (MRSA), and *Vancomycin-Resistant Enterococci* (VRE) were collected from 59% and 46% of the rooms in hospitals, respectively. Their presence was associated with insufficient surface cleaning and nurse understaffing. Moreover, the research shows that urinary catheters are responsible for more than 80% of infections of the urinary tracts [4,5]. Such a situation has prompted scientists to research the effectiveness of increasing the antimicrobial activity of the polymers and looking for new agents to combat pathogenic organisms. 

The methods of creating polymeric materials which inhibit or kill microorganism are constantly being sought. These materials can be produced by a direct mixing of the biocide agent with the polymer during processing (e.g., extrusion) or by applying a coating on the surface of the protected object. The metal particles such as silver and copper are the most commonly used additives. Silver shows high antimicrobial activity, and it is widely used in the packaging industry since it improves the shelf life of food [6]. However, some research confirms that silver ions and silver nanoparticles kill bacteria, human mesenchymal stem cells, and peripheral blood mononuclear cells at the same concentration. Due to the release of ions, silver ions can interact with cell wall components, nucleic acids, or metabolic enzymes. However, some other papers show that the antimicrobial mechanism is based on not direct but indirect damage caused by reactive oxygen species and the subsequent oxidative stress [7,8]. It is important to highlight that other metal particles such as copper possess a lower price and high antimicrobial properties against many types of bacteria, and therefore it is used as a water purifier, antibacterial and antifouling agent. To avoid working with expensive metal particles, metal oxides (e.g., CuO) are commonly used instead of silver salts such as silver nitride and silver acetate. In the group of metal oxides, titanium dioxide (TiO_2_) and zinc oxide (ZnO) have been tested as a filler in the polymers to obtain the biocide material. The first one has odor inhibition, a self-cleaning mechanism and low price [9], while ZnO enhances reactive oxygen species responsible for the bacterial inhibition mechanism [10]. It has been tested as an effective biocidal material for the protection of old paper documents [11]. From the other group of biocidal additives, aluminum (Al), iron oxide (FeO), magnesium oxide (MgO), calcium oxide (CaO), carbon nanotubes (CNTs), and even montmorillonite have been used as fillers in the different thermoplastic polymers. The examples of biocidal composites based on thermoplastic polymers are included in Table 1. As it can be seen from the collected data, metal-based additives are still primarily used as biocidal materials. 

It should be noted that the antimicrobial activity of the metals and their oxides is not always as good as desired. The biocide composites are often resistant to only some and not all strains of bacteria, fungi, or viruses. It is so due to the too long production–consumption cycle of particles, which lowers their efficiency and relatively small surface area [12]. Hence, to improve the biocidal properties of the metals and their oxides, it has been started to synthesize them with the nanoscale using the established processes in the recently developed nanotechnology science. As a result, the nanoparticles having new physicochemical properties are formed. Compared to microparticles, their surface-area-to-volume ratio is much higher, affecting the significant increase in biological activity. Because of the higher activity of the nanoparticles themselves and resulting faster dissolving in a solution, more metal ions are formed that interact with the pathogens [2]. 

Many different approaches have been tested to incorporate the biocide nanoparticles in the polymers, and they are mainly dependent on the final applications of the material (nanocomposite) as well as the possibilities and limitations of the method. For instance, the biocidal polymeric fibers can be produced by immersing the pure fibers in the nanoparticles solution or through the melt-spinning process, which does not require using any solvent. The main drawback of the nanoparticles is their tendency to form agglomerates, the presence of which may reduce the final properties of the material. Therefore, they should be destroyed during manufacturing to obtain the homogenous dispersion and distribution of the nanoparticles in the whole polymer matrix. The twin-screw extrusion (melt-blending) is often applied for the mixing of thermoplastics with nanoparticles, since the applied shear rate causes agglomerates destroying, resulting in homogenous dispersion of the nanoparticles [13]. The biocidal nanocomposites in the form of pellets or powder can be further applied in the processes such as injection molding (containers, syringes), melt-spinning (fibers, yarns), single-screw extrusion (filaments for 3D printing), or melt-blown (masks, filters). 

**Table 1:** The examples of antimicrobial thermoplastic composites described in the literature.

Polymer Matrix	Filler Type and Concentration	Pathogen	Activity	Ref.
PA12	2 wt % PHMG-DBS	*Trychophyton mentagrophytes*	qualitative activity	[14]
PA12	2–3 wt % silver nanopowder	*Escherichia. coli* *Staphylococcus aureus*	qualitative activity	[15]
PA12	4 wt % cuprous oxide-Cu_2_O	*E. coli* *S. aureus*	qualitative activity	[16]
PA11	5 wt % PHMG-DBS	*E. coli* *Bacillus subtilis*	>99.9%	[17]
PA11	10 wt % Cu	*E. coli*	>99.9%	[18]
PLA	1–5 wt % montmorillonite	*S. aureus* *Enterococcus. faecalis* *E. coli*	lack of activity	[19]
PLA	8 wt % TiO_2_	*Aspergillus fumigatus* *E. coli*	>94%	[17]
PANI	1 wt % and 3 wt % AgNO_3_	*S. aureus*	over 99%	[20]
PP	TiO_2_ coated with 1 wt % of nano-Ag	*S. aureus*	over 99%	[3]
PP	5 wt % TiO_2_-nanotubes	*E. coli*	17%	[21]
LDPE	10 wt % Organo Modificated CaO	*E. coli*	99.9%	[22]
PAN	3 wt % AgNO_3_/3 wt %TiO_2_	*S. aureus*	over 99%	[23]

PA—Polyamide, PLA—Poly(lactid acid); PANI—Poly(aniline); PP—polypropylene; PAN—poly(acrylonitrile); PHMG-DBS—poly(hexamethylene guanidine) dodecylbenzenesulfonate.

As confirmed in much research, nanoparticles are responsible for the antimicrobial properties in the polymer/metal nanocomposites. However, their effectiveness is dependent on the number of metal ions formed in the presence of water. According to the mechanism described by Palza for the bacteria [2], water molecules coming from the bacteria medium diffuse into the surface of the nanoparticles. Then, water and dissolved oxygen cause the dissolution or corrosion processes when they reach the nanoparticles’ surface, leading to the formation of the ions. Moving metal ions reach the composite surface, damage the bacteria membrane and diffuse into the pathogen. 

It should be noted that the biocidal properties of the polymer nanocomposites are dependent on the type of polymer used, especially its polarity and crystallinity. The highest number of metal ions is formed in more polar (hydrophilic) polymer matrices having lower crystallinity. It is associated with the ease of water absorption and movement of the macromolecules chains, which do not prevent reaching the surface of the water molecules [24].

This paper presents the characterization of the polymer/metal nanocomposites based on PA12 and the mixture of the nanoparticles of metal oxides-CuO, ZnO, and TiO_2_ obtained by the melt-blending process. Such composites containing different types of metal oxides have not yet been described in the literature, which emphasizes the high novelty of the conducted research. It was examined how much the metal oxides affect the rheological and thermal properties of PA12, and the results were related to the polymer wettability and dispersion of the nanoparticles. The biological tests included the activity against representative bacteria (*Escherichia coli*), fungi (*Candida albicans)*, and viruses (*Herpes simplex* virus type 1 and *Adenovirus 5*). Additionally, in vitro cytotoxicity of the nanocomposites and the programmed cell death were assessed. Because most of the polymer/metal nanocomposites described in the literature are tested against only one type of pathogens (see Table 1), the presented research is valuable for current challenges with the high activity of the microorganisms. 

## 2. Materials and Methods

### 2.1. PA12 Composites Preparation

As a polymer matrix, PA12 was supplied in pellet form from the Evonik company (Essen, Germany), with the trade name Vestamid Z7321. It has the density of 1.01 g/cm^3^, water absorption of 1.5%, a melting point of 179 °C, and a processing temperature between 190 and 240 °C. The pellets of PA12 were initially dried in a vacuum oven at 90 °C for at least 24 h before further usage. As an antimicrobial filler, a mixture of CuO, ZnO, and TiO_2_ powders with the trade name ACRAZ-172T was used as a filler. It is in the form of a light black powder. The average grain size of these oxides was between 300 and 1000 nm, a melting point of 962 °C, and a boiling point of 2200 °C. Three oxides were intentionally applied as a filler to PA12 to strengthen antimicrobial activity of the composite. Copper oxide shows high antimicrobial activity at its surface. Zinc oxide is much less toxic, but when in contact with copper oxide, the system shows much stronger activity. Titania is added to the composite to increase wettability of the surface, which is necessary for proper and high activity of CuO and ZnO.

**Figure 1:**
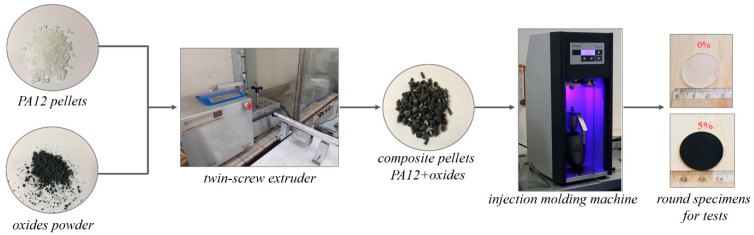
The approach used for the nanocomposite manufacturing.

As presented in Figure 1, PA12 composites were produced by mixing the polymer pellets and the oxides powder using a laboratory twin-screw extruder HAAKE MiniLab (ThermoFisher Scientific, Waltham, MA, USA) equipped with a bypass cycle. The concentration of the metal oxides was 1, 2, 3, 4, and 5 wt %. The composites were extruded at 190 °C, with the rotations of the screws of 100 rpm and a mixing time (residence time) of 5 min. The fabricated pellets were afterwards processed into the round specimens with 25 mm diameter and 0.1 mm thickness using a HAAKE Mini Jet Piston Injection Molding System (ThermoFisher Scientific, Waltham, MA, USA). The parameters of the injection molding were as follows: 220 °C—the temperature of the barrel, 50 °C—the mould temperature, 700 bars and 10 s injection pressure and time, and 600 bars and 8 s—the post-processing injection pressure and time. The produced rounds were used in the rheological and antimicrobial tests as well as for the microstructure observations. Using a computer microtomography, it was also determined that the pores content in the composites was lower than 2%.

### 2.2. The Analysis of the Physicochemical Properties

The wettability of the metal oxides by PA12 pellet grains was analyzed using a Drop Shape Analyser apparatus DSA100M(Kruss GmbH, Hamburg, Germany) equipped with a high-temperature cell and a digital camera (20 frames per second). The temperature cell enables the heating of samples in the range from 0 to 400 °C and controls the temperature inside the cell with the accuracy of 0.1 °C. Wetting angles were measured by tangents fit. In order to perform the measurement, the PA12 pellet grain was placed on the surface of the oxides powder filler in the center of the cell under room temperature conditions. Then, the heating of the cell was started at a rate of about 10 °C per minute. Thanks to the continuous video observation during the experiment, changes in the texture of the pellet matrix during its melting and interaction with the metal oxides were observed. The first melting effects of the PA12 pellet, the first contact angle of the molten pellet and contact angles for its full possible spread were also noticed. 

The surface composition and electronic states of the elements at the surface of the filler patty before and after contact with melted PA12 were analyzed with X-ray photoelectron spectroscopy (XPS). The EA-15 (PREVAC, Rogów, Poland) hemispherical analyzer equipped with dual anode X-ray source RS 40B1 (PREVAC, Rogów, Poland) was used for the surface analysis. The spectrometer was calibrated with Ag, Au and Cu foils according to the ISO 15472:2010 standard. The area of analysis was approximately 2 mm^2^ and the depth of analysis was about 10 nm. 

The measuring of the quality of the composites and dispersion of the oxides in the PA12 matrix was carried out using a scanning electron microscope (SEM HITACHI TM3000, Tokyo Japan). The samples for the test were prepared by freezing the specimens after injection molding in liquid nitrogen for 10 min and then breaking them. The samples were stuck to the metal measuring tables using double-sided carbon tape so that the fractures faced upwards. The surfaces of the samples were coated with a conductive layer using an electro-deposition method to make them electrically conductive. The microstructure images were collected at magnifications of ×500, ×1000, ×1500, and ×2000. The quantitative analysis of the oxide dispersion was performed using the ImageJ software (Version: 2.0.0, National Institutes of Health and the Laboratory for Optical and Computational Instrumentation, Madison, WI, USA), which allows to count the diameters of agglomerates. For each composition, a minimum of seven images was taken for the calculation. The obtained results were shown in the form of histograms. 

Thermogravimetric analysis (TGA) was performed to analyze the thermal stability of the composites. This analysis was carried out using a TGA Q500 (TA Instruments, New Castle, DE, USA) for the samples weighing 10 ± 0.5 mg placed in platinum pans. The samples were heated from 0 to 820 °C in a nitrogen atmosphere with a heating rate of 10 °C/min and a flow rate of 10 mL/min in a chamber and 90 mL/min in an oven. From the obtained curves, the degradation temperature at 5% (T_5%_) and 10% (T_10%_) weight loss, and the temperature of the maximum weight loss rate (T_d_), were determined. 

The effect of the addition of the metal oxides on the thermal properties of PA12 was studied using a Q1000 Differential Scanning Calorimeter (TA Instruments, New Castle, DE, USA). The samples weighing 6.5 ± 0.2 mg were placed in an aluminum hermetic pan. The used program included first heating from −80 °C to 240 °C, then cooling from 240 °C to −80 °C and second heating from −80 °C to 240 °C with a scan rate of 10 °C/min at nitrogen atmosphere. Using the Universal V4.5A TA software, the characteristic temperatures such as melting point (*T_m_*) and crystallization temperature (*T_c_*) were determined. The crystallinity content (*X_c_*) of the PA12 composites were calculated from the following equation number 1:

(1) $$X_{c}\left(\%\right)=\frac{\Delta H_{c}}{\Delta H_{m}^{{}^{\circ}}\left(1-x\right)}\cdot 100\%$$

where:

Δ*H_c_* is the enthalpy of melting taken as the area under the melting peak from the second heating curve,
Δ$H_{m}^{{}^{\circ}}$ is the melting enthalpy of 100% crystalline PA12, which is 209 J/g [25]
*x* is the weight fraction of metal oxides.

The processability of the PA12/oxides composites was investigated in terms of their rheological properties such as complex viscosity, storage, and loss modulus. The round specimens having a diameter of 1.5 mm and a thickness of 2 mm (Figure 1) were tested using an oscillatory ARES 4400-0107 rheometer (Rheometric Scientific Inc., TA Instruments, New Castle, DE, USA) in a parallel plate geometry mode. Firstly, the amplitude sweep test as a function of the variable strain γ (0.07–100%) at a constant frequency of 1 Hz was performed. From the linear elastic range of the storage modulus curves, the strain of 10% was selected. With that strain, a dynamic oscillatory stress-controlled rotational test was performed at 190 °C with a frequency sweep from 0.1 to 100 Hz.

### 2.3. Biological Tests

The prepared composites in the form of the round specimens were analyzed against representative bacteria, fungi, and viruses using the round from the injection molding machine (see Figure 1). The antibacterial activity of samples against *E. coli* was determined using two methods. The qualitative determination was made based on the modified protocol of ISO 20645 and the quantitative determination was based on the AATCC Test Method 100 protocol with modification. In the ISO 20645 method, the samples were placed between two agar layers and the plates were kept for incubation at 37 °C for 24 h. The lower layer contained 10 mL of Luria broth (LB) and the upper layer had 5 mL of LB with 5 × 10^5^ cells/mL of the bacteria. The bacteria came from a previous LB inoculum incubated for 24 h at 37 °C. At the end of incubation, the zone of inhibition formed around the round specimen was measured in millimeters and recorded. In the AATCC Test Method 100, the tested microorganisms were grown in LB at 37 °C for 24 h, and then they were suspended in appropriate media and the cell densities were adjusted to 0.5 McFarland standards at 630 nm wavelength using a spectrophotometric method (1–1.5 × 10^8^ cells/mL). The samples were then inoculated with 5 mL microbial suspension and incubated for 24 h. After that contact period, 45 mL of neutralizing solution (phosphate-buffered saline PBS, pH = 7.4) was added to the falcon tubes containing the inoculated treated swatches. After 1 min of shaking, standard serial dilution was performed and 10 μL of each solution were cultured on LB plates and incubated for 24 h at 37 °C. The reduction of microbes was calculated by the following equation: [(no. bacteria on pure PA12—no. bacteria on PA12 with oxides)/no. bacteria on pure PA12] × 100.

Antifungal activity of PA12 was assessed using the following quantitative methods: AATCC TM100-2019 (The American Association of Textile Chemists and Colorists, AATCC TM100-2019, “Test Method for Antibacterial Finishes on Textile Materials: assessment of” and PN-EN ISO 20743:2013-10E “Textiles—Determination of Antibacterial Activity of Textile Products”). Briefly, *C. albicans* ref. strain 90028 ATCC (LGC Standard, Poland) at 5 × 10^3^ to 6 × 10^5^ CFU/mL of YEPD was incubated with swatches at 35 °C for 24 h. Then, tenfold dilutions were plated on YEPD agar and the number of CFU was counted for tested and control (without metal oxides) swatches. Additionally, anti-*C. albicans* 90028 activity was performed using the qualitative method AATCC TM90-2011 (2016)e (The American Association of Textile Chemists and Colorists, AATCC TM90-2011(2016)e, “Test Method for Antibacterial Activity of Textile Materials: Agar Plate”). Briefly, the tested and control swatches were put on top of the solid agar inoculated with blastoconidia (2.6 × 10^3^ CFU/mL) and incubated at 35 °C for 24 h. Zones of inhibition and growth under the tested swatches were compared to the control ones. R% was calculated using Equation: (A − B)A × 100% = %R, where A means CFU recovered from the PA12 control inoculated with *C. albicans* and incubated over the 24 h contact period; B means CFU recovered from the PA12 with metal oxide inoculated and incubated over the 24 h contact period.

Virucidal properties were determined using Herpes simplex virus type 1 (HSV-1-ATCC^®^ VR-1493™) and human *Adenovirus 5* (Ad-5 virus—strain Adenoid 75, ATCC VR-5^TM^). Two types of cell lines obtained from American Type Culture Collection—ATCC (Rockville, MD, USA) (A549-human lung carcinoma (ATCC, No. CCL-185^TM^) and HeLa -human cervix carcinoma (ATCC, No. CCL-2^TM^)) were used in this experiment. Dulbecco’s Modified Eagle’s Medium—DMEM (Lonza, Basel, Switzerland) served as substrate. Media were supplemented with 10% fetal bovine serum (FBS) and 4 mM L-glutamine (Biological Industries, Kibbutz Beit-Haemek, Israel), 100 U/mL of penicillin and 100 g/mL of streptomycin (Sigma-Aldrich, Munich, Germany). The substances were tested using International Standard ISO 21702 (Measurement of antiviral activity on plastics and other non-porous surfaces, Geneva, Switzerland, 2019). This document specifies proper methods for measuring antiviral activity on plastics and other non-porous surfaces of antiviral-treated products against specified viruses. According to the standard (ISO 4.1), alternative viruses to influenza virus and feline calicivirus may be used. Therefore, the study was conducted using *Adenovirus 5* commonly found in humans. This pathogen represents non-enveloped viruses, belonging to the group of most difficult to inactivate viruses. The virus included in the test is responsible for colds. The second study was conducted using human herpesvirus type 1. This pathogen represents enveloped viruses. The virus included in the test is responsible for herpes in human. In the laboratory, these strains reach high titers, so it was possible to validate the method quickly. The virucidal efficacy was tested according to ISO 21702:2019 (ISO 7. 1-7. 4). For this purpose, a sample of the test material (ISO 4. 3. 16) treated with SCDLP neutralizer was placed in a sterile petri dish. On the sample prepared in this way, 400 µL of the test virus were spotted and covered with a 2 × 3 cm layer of polymer. The sample was incubated at 25 °C for 24 h at 90% humidity. After this time, the virus was collected by pipette. The study was conducted in 3 replicates. In parallel, the whole procedure was repeated using a control specimen (pure PA12) as a non-virulent material. In addition, immediately after virus inoculation onto the control round specimen, virus titers were tested (3 replicates). According to ISO 21702:2019, which allows to use the TCID_50_ method instead of the plaque test, serial dilutions up to 10^−8^ of the viruses collected from each disc were prepared. In eight repeats, 50 µL of each dilution were added to the microtiter plate containing a monolayer of confluent A549 or HeLa cells. The plates were observed daily for up to 4 days for the development of viral cytopathic effect, using an inverted microscope (Olympus Corp., Hamburg, Germany; Axio Observer, Carl Zeiss MicroImaging GmbH). The calculation of the infective dose TCID_50_/mL was conducted using the Spearman and Karber method with the following formula:

log_10_TCID_50_ = *x*_0_ − 0.5 + Σ*r*/*n* (2)

where:

*x*_0_ is log_10_ of the lowest dilution with 100% positive reaction,
*r* is the number of positive determinations of lowest dilution step with 100%,
positive and all higher positive dilution steps,
*n* is the number of determinations for each dilution step.

The programmed death of the L929 ATCC cell line (LGC, Standard, Poland) treated with PA12/metal oxides composites was assessed using flow cytometer BD FACS Lyrics 2L6C with FAC Suite Software 1.4 RUO (BD Biosciences, Mississauga, ON, Canada). Liquid extracts of PA12 containing 3 wt % and 4 wt % oxides were prepared in DMEM (ATCC, LGC Standard, Poland) after 4 h and 24 h extraction according to ISO 10993-5:2009 (E). The monolayer of cells (1 × 10^4^ cells/mL) was treated with the extracts for 18 h. Then, the treated cells were stained with annexin V and propidium iodide (FITC Annexin V Apoptosis Detection Kit I BD Biosciences, Franklin Lakes, NY, USA) [26]. Briefly, L929 were seeded in 24-well plates (10,000 cells/mL) and incubated for 24 h. Then, the medium was replaced with 100 μL of extracts (10% or 100%). The plates were incubated for 24 h at 37 ± 1 °C with 5% CO_2_. Simultaneously, to assess in vitro cytotoxicity (ISO 10993-5:2009E) of PA12 containing 3 wt %, 4 wt % and 5 wt % oxides, L929 (8000 cells/100 µL) were seeded in the 96-well plates. After treatment, 11 µL PrestoBlue reagent (ThermoFisher Scientific, Waltham, MA, USA) was directly put into each well and incubated for 1.5 h. The absorbance was recorded at 570 nm (and reference at 600 nm) with Infinite M200 reader (Tecan, Durham, NC, USA). The GraphPad Prism 8.4.3 (GraphPad Software Inc., La Jolla, CA, USA) was used for the data analysis. Differences between the groups were tested using Bonferroni’s test multiple comparison tests. Differences were considered statistically significant at *p* < 0.05.

## 3. Results

### 3.1. Contact Angle Measurements 

**Figure 2:**
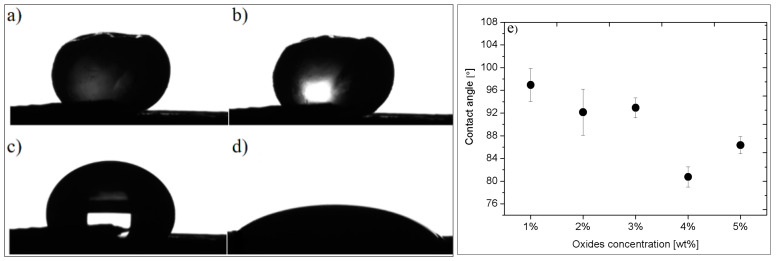
Images of the PA12 pellet melted at the filler surface: (**a**) original PA12 pellet; (**b**) first softening of the pellet at 150 °C; (**c**) completely melted PA12 at 180 °C; (**d**) wetting of the filler tablet by fully spread PA12 polymer at 224 °C; (**e**) dependence between contact angle of in PA12 composites and metal oxides concentration.

The wettability of the metal oxides by melted PA12 was analyzed from the images taken during the melting process in Figure 2. In a model system, the filler was used in the form of the tablet contacted with a polymer pellet. The tablet was heated, and the shape of the pellet was analyzed. PA12 polymer starts to melt at the temperature of about 150 °C (Figure 2b) and is entirely molten at 180 °C (Figure 2c). However, the full spread of the molten polymer over the filler surface occurred only at the temperature of 224 °C (Figure 2d). Carbonization of the melted polymer was observed above 275 °C. The influence of the oxide concentration on the observed contact angles of the molten material is presented in Figure 2e. As seen, the increase of the oxide content in the filler leads to diminishing the contact angle of the molten pellet. It is worth remembering that PA12 is the example of the hydrophilic polymer which is easily wettable by water. However, in the presence of the hydrophobic metal oxides, the composites of PA12 become less hydrophilic. This effect is enhanced at higher concentration, especially at 4 wt %, for which the contact angle is 80°. The further increase of the oxide concentrations reverses the observed effect.

### 3.2. Surface Studies 

**Figure 3:**
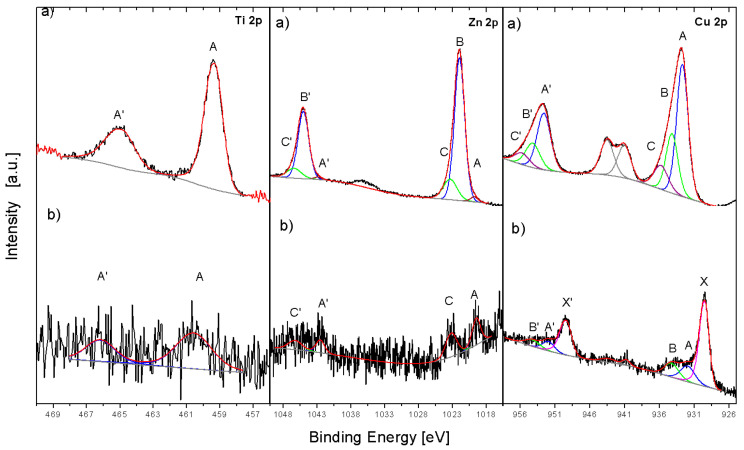
XPS analysis of the filler before (**a**) and after (**b**) interaction with PA12. The comparison of Cu 2p, Zn 2p and Ti 2p spectra.

An interaction of PA12 with the surface of oxide filler was studied. The surface of the oxide filler powder before and after contact with melted PA12 was analyzed by XPS technique. Table 2 shows concentration of the elements at both studied surfaces. The results indicate that the filler surface is strongly covered by polymer containing carbon and a small amount of nitrogen. The amount of copper (Cu), zinc (Zn) and titanium (Ti) after contact with melted PA12 at the interface filler/polymer was low. Moreover, the electronic state of the metals after reaction with melted PA12 changed, indicating reduction of the oxides at the interface (Figure 3).

In the case of Cu 2p excitation, the filler (Figure 3a) contains mainly CuO (A component), which surface is hydroxylated (B) and covered with a small amount of organic adsorbate (C) [27]. The contact with melted PA12 revealed in Cu 2p spectrum (Figure 3b) that C component disappeared and new X component appeared, which is assigned to reduction of CuO to Cu in strongly nucleophilic surrounding, and suggests strong interaction of copper oxide components with amide groups of PA12. In the case of the Zn 2p line, the filler contained mainly ZnO (B component) with hydroxylated surface (C component) and with some zinc alloy (possibly Zn-Cu bonding) surface compound (X component [27]. After the contact with melted PA12, ZnO-related components disappeared in the spectrum, showing only the alloy (X) and hydroxylated (B) ones. This can indicate strong polymer adsorption at the ZnO surface with the exposition of uncovered surface species. The described changes in the spectra of Cu, Zn and Ti in the filler indicate strong interaction of the amide groups coming from the PA12 macromolecules with metal cations. It leads to partial reduction the filler surface (metallic copper found), selective bonding with polymer (titania covered by polymer without surface change), or bonding to amide groups (zinc oxide).

### 3.3. Microstructure 

To realize the potential of the functional fillers fully, it is necessary to obtain the well-dispersed and homogenously distribute particles in the whole polymer matrix. Therefore, the morphologies of PA12/metals oxides composites were analyzed by SEM on pellets just after extrusion and in the specimens after injection molding process. Table 3 presents the SEM images of the pellets where white areas refer to the metals oxides and they are well-dispersed in the PA12 matrix. A few agglomerates of the metals oxides can be observed in some places, which are quantitatively determined by calculation of their average grain size. It is clearly seen that increasing the content of the metals oxides causes decreasing the agglomerates diameter from 24.8 µm for 1 wt % to 4.29 µm for 5 wt % of the filler. Looking into the diameters of the initial metal oxides powder, which range from 0.3 to 1 µm, the observed agglomerates can be called as secondary ones since they are formed during extrusion from the primary agglomerates occurring in powder form. Such a scenario takes place in low viscous polymers where macromolecules move freely and when a shear rate is too low to destroy the formed agglomerates [28]. Therefore, decreasing the agglomerates sizes linearly with the oxides concentration is associated with the increase of viscosity of the PA12 matrix and higher shear force caused by rotating screws during extrusion. A similar analysis was made for the injection-molded specimens and the measured agglomerates are included also in Table 3. It can be seen that there is the same tendency as for the pellets. At higher metal concentration, the agglomerates diameters are smaller, but slightly bigger than determined for the pellets. In that process, the pellets are again melted and subject only to the pressure. Hence, the agglomerates are not able to be destroyed but they can form the new, secondary agglomerates. 

### 3.4. Thermal Properties 

**Figure 4:**
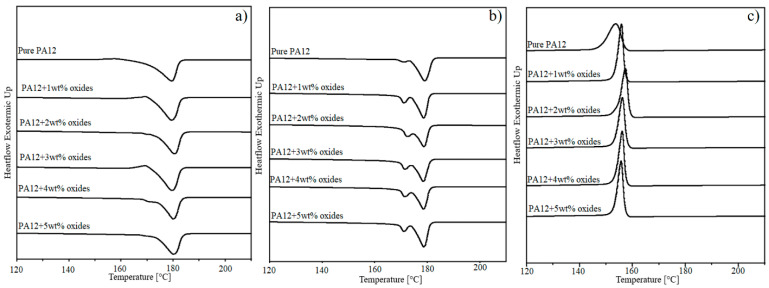
DSC curves for studied materials; (**a**) first heating; (**b**) second heating; (**c**) cooling.

The thermal stability of the PA12 composites was determined using thermal gravimetric analysis. The results are presented in Table 4 and the raw TGA curves for each composite are collected in the Supplementary Data (Figures S1–S6). The results indicate the deterioration of the thermal properties of the composites with the increase in the amount of metal oxides. The temperature of the 5% material weight loss decreases of about a maximum of 7 °C at the highest oxides concentration in comparison to pure PA12. In the case of 10% weight loss and the fastest material degradation, the effect of the oxides is negligible. A weak impact of the oxides on the thermal stability can be connected with the presence of the oxides agglomerates. DSC test was performed to identify the effect of the metal oxides on the melting and crystallization behavior. The results were collected in Figure 4 and Table 4. For some of the composites, the glass transition decreases by about 1–5 °C without linear dependence with the oxides content. This is associated with the blocking of the polymer chains by the filler, which is more effective for the homogeneously dispersed oxides. During the 1st heating (Figure 4a), all materials show a single melting peak with a melting point at 180 °C, which refers to the presence of γ crystal forms. There is no change in the melting point indicting the weak nucleation effect of the added oxides visible also by small changes in the crystallinity content. On the thermogram obtained during the 2nd heating, a single melting peak splits to the double one (Figure 4b), suggesting the formation of the second crystalline phase-α. That crystal phase melts at the lower temperature around 165 °C and it is formed as an effect of a slow cooling subjected after the 1st heating cycle. Such a scenario favours the parallel arrangement of the polymer chains characteristic for the α phase. Because the melting peak obtained during the 2nd heating has a shoulder, it confirms the partial deformation of the α-form crystals structure to the γ-form [29,30]. That transformation is probably hindered in the presence of the metal oxides because the composites have lower crystallinity content than neat PA12. Moreover, the crystallinity changes only about few degrees with increasing amount of metal oxides, similarly to the crystallization temperature, which is not influenced by the addition of the oxides. However, it can be seen that peaks coming from the composites are narrower than for neat PA12. This fact shows the presence of more homogenous crystal phase. Similarly, the addition of copper spheres and copper flakes to PA12 do not affect characteristic temperatures of the polymer determined from the DSC thermograms [31]. 

### 3.5. Rheological Properties 

**Figure 5:**
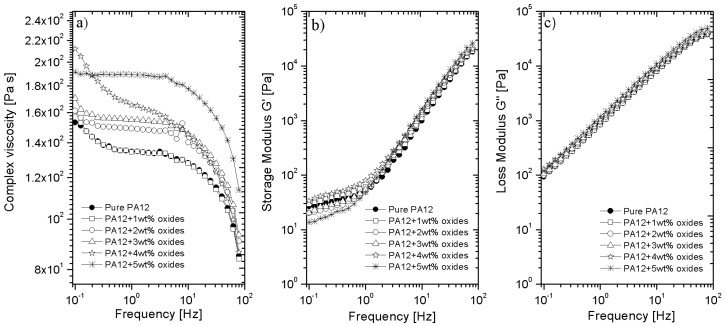
(**a**) Dynamic viscosity; (**b**) storage modulus G′ and (**c**) loss modulus G″ as a function of frequency for pure PA12 and PA12 with the addition of 1–5 wt % metals oxides.

The assessment of the rheological behavior of the composites is important regarding their processing performance and interactions of the polymer with the added fillers. Using rotational rheometer, the effect of the metal oxides addition on the viscosity, storage and loss modulus were analyzed. Figure 5 presents the curves obtained at 190 °C, which are presented in a logarithmic scale as a function of the angular frequency. The strain was selected as 10% from the linear viscoelastic range, and it was used in all of the tests. In the measured frequency range, for all materials including neat PA12, the viscosity curves have the linear dependence but only at low frequencies (Figure 5a). The further increase the frequency causes decreasing the viscosity, which is characteristic for the shear-thinning pseudoplastic liquids [32]. The lack of typical Newtonian curve depicted for pure PA12 can be associated with the changes in the molecular weight, the presence of the gas bubbles or additives, especially plasticizers [33]. The viscosity is higher for the composites containing 2–4 wt % of metal oxides in comparison to the viscosity of pure PA12. Because for PA12 + 1 wt % oxides the viscosity has the same characteristic for the neat PA12, it can be stated that metal oxides inhibit the movement of the polymer chains at approximately 2 wt %. Despite the fact that the composites have higher viscosity, such an increase is not significant. For instance, pure PA12 has the viscosity at 1Hz equals to 135 Pa·s, while for the composite with 5 wt % oxides, the viscosity is 190 Pa·s. In Figure 5b,c, the dependence of the storage (G′) and loss (G″) modulus refers to the elastic and viscous behavior of the material respectively. Both of the moduli increase together with the frequency and they are not dependent on the oxides concentration. So, it means that the interactions between PA12 and metal oxides are not as strong as for the reported composites of PA12 with, e.g., carbon nanotubes [34]. Obviously, such interactions are associated with a lower surface area of the used oxides, rather than with the dispersion of the oxides in the matrix. From the presented graphs, it can also be observed that for all materials, the loss modulus is larger than the storage modulus in the whole frequency range. This suggests that the studied composites exhibit more viscous than elastic properties. Looking from the practical point of view, such a behavior is desired for the processes in which PA12-containing metal oxides can be applied for the final products. The first example is a melt-blown process in which the non-woven fabric can be manufactured and used as protective masks or filters [35]. The other possibility is to use PA12 composites in the FFF (Fused Filament Fabrication) 3D printing process to print the medical components such as respiratory valves, face shields or even the implants from the medical grade of PA12 [15]. 

### 3.6. Biocidal Properties 

**Figure 6:**
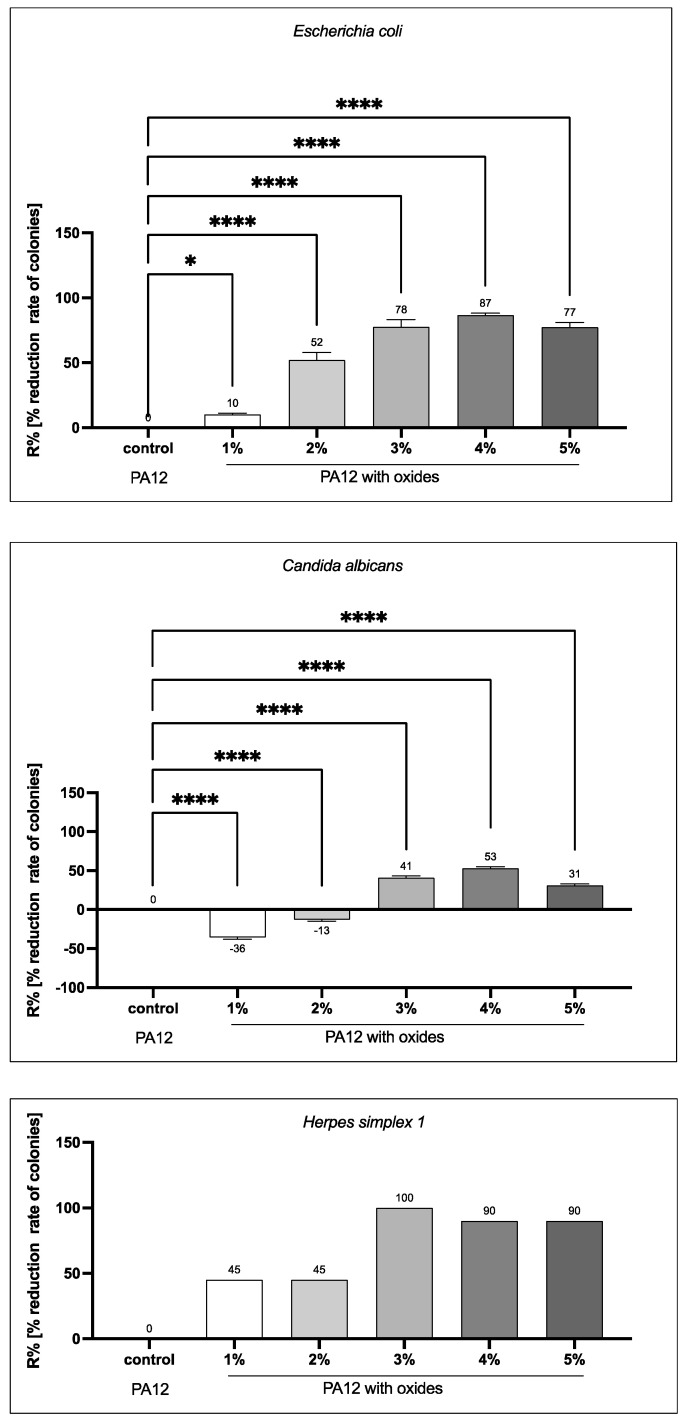
The antimicrobial activities in term of reduction (%) rate of colonies (CFU), against: *Escherichia coli*, *Candida albicans* and *Herpes simplex 1.* Differences with *p* < 0.05 were considered statistically significant. One asterisk (*) indicates *p* < 0.05, four asterisks (****) indicate *p* < 0.0001. Since the results for viruses are discontinuous, error bars, as well as one-way analysis of variance, cannot be added.

One of the objectives of this paper is to confirm the biocidal properties of PA12 as the effect of the addition of metal oxides to it. In order to meet this objective, the round specimens fabricated by injection molding (were tested against example bacteria, fungi and viruses. The qualitative activities are included in Appendix B, Figure A3 and Figure A4, while the quantitative analysis for all pathogens is shown in Figure 6. In the case of *E. coli*, the qualitative antimicrobial activities of the pure PA12 and its composites show no inhibition zone for all of the tested samples (Appendix B, Figure A3). However, the slight growth of bacteria under all materials with oxides was observed, which indicates low antibacterial activity. The quantitative antimicrobial activities (Figure 6) of the studied materials depict the reduction of the bacteria colonies as the effect of the metal oxides addition. As it can be seen in the graph, the highest CFU reduction rate (R%) against *E. coli* was 87% in PA12 containing 4 wt % oxides, whilst 5 wt % causes decreasing of the reduction rate. 

**Figure A1:**
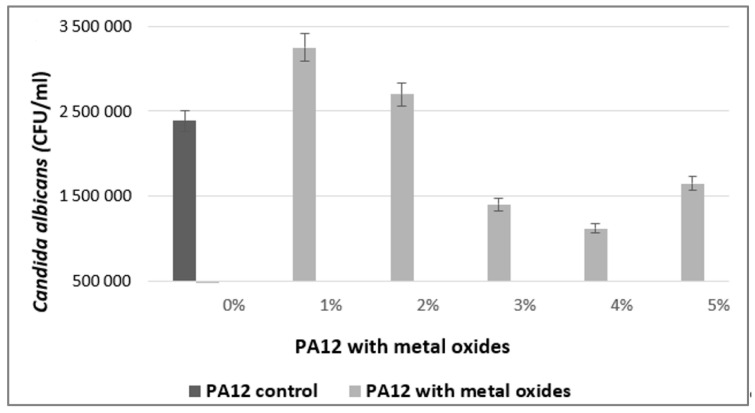
Antifungal activity of the PA12 with metal oxides assessed using the AATCC TM100-2019 quantitative method. Legend: The results obtained using AATCC TM100-2019 are valid due to: (1) “0” CFU of *C. albicans* recovered from the uninoculated swatches; (2) a significant increase in CFU (≥1 log) recovered from the PA12 control (without metal oxides) inoculated with *C. albicans* after 24 h was noted, and it was over the CFU recovered from the PA12 control without metal oxides at “0” contact time (immediately after inoculation).

**Figure A2:**
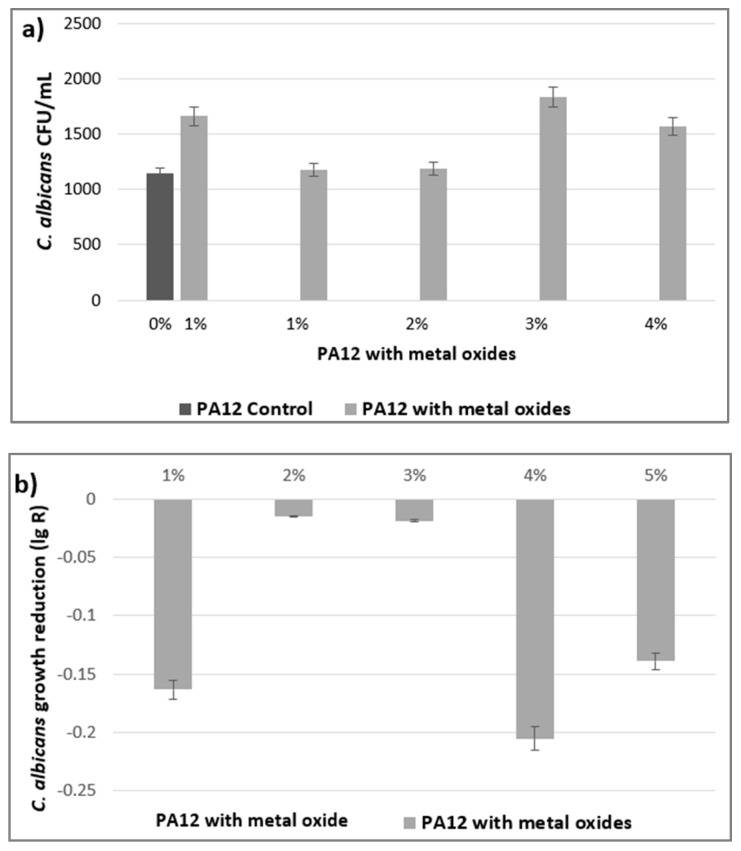
(**a**,**b**). Antifungal activity of the PA12 with metal oxides assessed using the PN-EN ISO 20743 2013-10E quantitative method.Legend: lg R was calculated using Equation: lg A − lg B = lg R, where A means CFU recovered from the PA12 control (without metal oxide) inoculated with *C. albicans* and incubated over the 24-h contact period; B means CFU recovered from the PA12 with metal oxide inoculated with *C. albicans* and incubated over the 24-h contact period.

**Figure A3:**
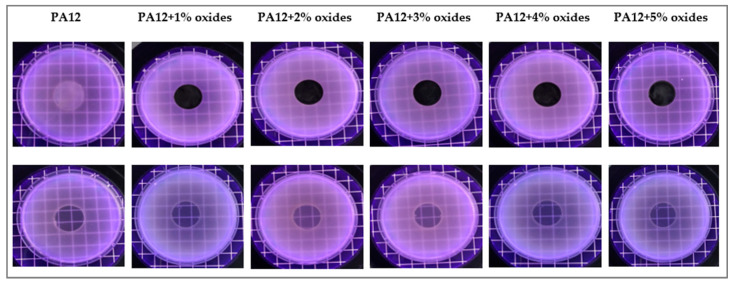
Comparison of *E. coli* growth in contact with PA12 and PA12 + 1–5 wt % oxides.

**Figure A4:**
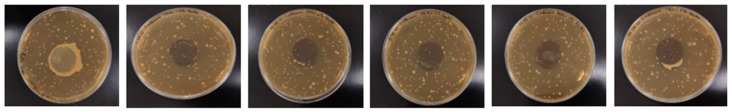
Fungistatic activity of the PA12 with metal oxides. *C. albicans* CFU on the YEPD agar growth medium inhibited under PA12 swatches contained 1, 2, 3, 4 and 5 wt % of metal oxides compared to the control PA12 without metal oxides. Visible confluent growth under the swatch as well as in proximity to the borders of a specimen.

The standard qualitative procedure clearly demonstrates the antifungal activity of PA12 containing metal oxide vs. the control PA12 (without metal oxides), showing the lack of this activity (Figure A1 in Appendix A). As it is shown in Figure 6, the most effective reduction rate of the *C. albicans*’ CFU was obtained for the composites with 3 wt % (R% = 41) and 4 wt % (R% = 53) of the metal oxides. PA12 displayed low antifungal activity in the suspension method according to PN-EN ISO 20743 2013-10E (see Figure A4 in Appendix B). Fungistatic effectiveness of the PA12 containing metal oxide was observed using AATCC TM90-2011(2016)e plate test (Figure A2 in Appendix A). The growth of *C. albicans* CFU was reduced under swatches containing metal oxides vs. the PA12 control (without metal oxides). There was no diffusion of antimicrobial agents from the swatches as indicated by the lack of a zone of inhibition (Appendix B, Figure A4). In the case of PA12 composites, the analyzed specimens displayed fungistatic activity. 

The activity of PA12 composites against Ad5 and HSV-1 within 24 h was also examined. Based on the results, there was no reduction in Ad 5 titer by any of the materials tested, but in the case of HSV 1 virus, the test material showed virucidal properties (Figure 6). After the exposure time, the virus titer was reduced compared to the control material-pure PA12. The highest activity was obtained for 3 wt % of the metal oxides with the reduction R% = 100. For 4 wt % and 5 wt % the reduction was slightly lower (R% = 90) and did not vary for the oxides concentration. PA12 composites, especially at concentrations of 3 wt %, but also at 4 wt % and 5 wt %, are capable of inactivating enveloped herpes viruses. So, it can be assumed that at these concentrations PA12 composites will be equally effective in inactivating other enveloped viruses belonging to the family of *Orthomyxoviridae* (human and animal influenza viruses), *Coronaviridae* (including SARS-CoV), *Flaviviridae* and *Poxviridae*, and blood-borne viruses such as HBV, HCV and HIV.

**Figure 7:**
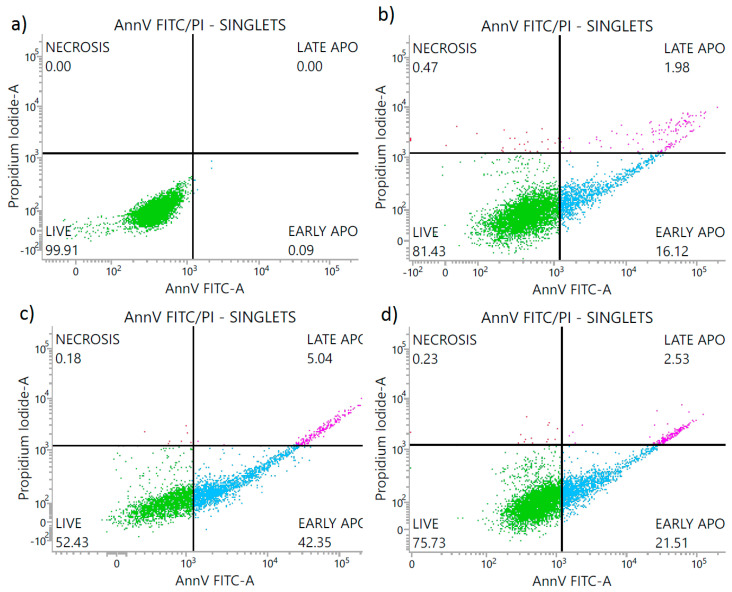
Percentage share of the L929 cells: alive, early apoptotic, late apoptotic and necrotic after 24 h treatment with: (**a**) DMEM—control group; (**b**) 4 h extract of pure PA12; (**c**) 4 h extract of PA12 with 3 wt % of metal oxides; (**d**) 4 h extract of PA12 with 4 wt % of metal oxides. The experiment was performed in triplicate.

**Figure 8:**
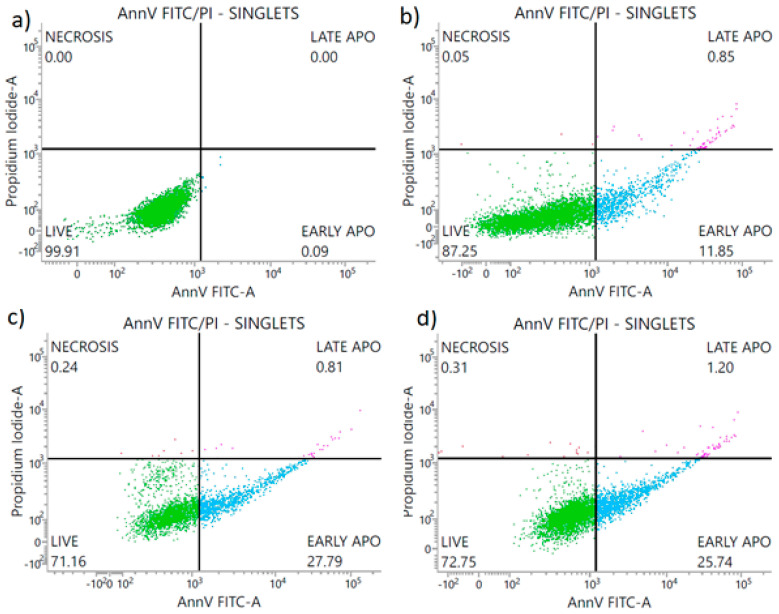
Percentage share of the L929 cells: alive, early apoptotic, late apoptotic and necrotic, after 24 h treatment with: (**a**) DMEM—control group; (**b**) 24 h extract of pure PA12; (**c**) 24 h extract of PA12 with 3 wt % of metal oxides; (**d**) 24 h extract of PA12 with 4 wt % of metal oxides. The experiment was performed in triplicate.

**Figure 9:**
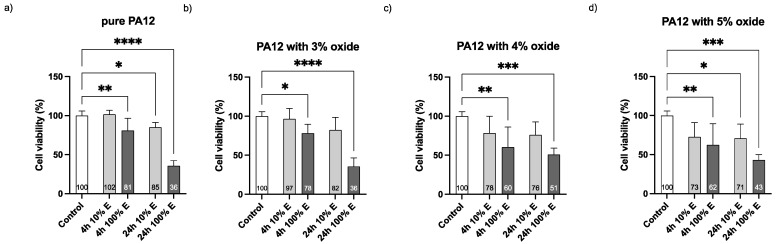
Cell viability: MTT assay. Histograms represent the percentage, with respect to control cells (Ctrl, 100%) of viable cells after 24 h treatment with 4 h and 24 h extracts at 10 and 100% concentration from (**a**) pure PA12; (**b**) PA12 with 3 wt % of metal oxides; (**c**) PA12 with 4 wt % of metal oxides; (**d**) PA12 with 5 wt % of metal oxides. Differences with *p* < 0.05 were considered statistically significant. One asterisk (*) indicates *p* < 0.05, two asterisks (**) indicate *p* < 0.01, three asterisks (***) indicate *p* < 0.001, four asterisks (****) indicate *p* < 0.0001.

As mentioned in the introduction, silver particles have been proven to be toxic to the human cells. Therefore, the new bioactive fillers are being researched to be used in protective materials for humans. Hence, to determine the mode of cell death generated by the PA12 composites, the Annexin V/FITC assay using flow cytometry was conducted and it was followed by the cytotoxicity assay. The treated fibroblasts displayed the features characteristic of apoptosis (Figure 7 and Figure 8). The Annexin V binding showed the loss of membrane asymmetry and phosphatidylserine (PS) exposure in fibroblasts treated with the PA12 containing 3 wt % or 4 wt % of metal oxides (Figure 7 and Figure 8). The fibroblasts treated with the extracts of PA12 (0–4 w% of metal oxides) underwent early apoptosis (11.85–27.79%), late apoptosis (0.81–5.04%) and necrosis (0.18–0.31%) respectively. So, the concentration of 3 wt % compared with 4 wt % generated a 2-fold higher percentage of apoptotic cells. In the case of fibroblasts, the composites containing 3 wt % of metal oxides in 4 h extract solution (Figure 7), significantly reduced fibroblast viability (52.43% of viable cells) vs. the 24 h extract (viability at 71.16% in Figure 8). For the first time PA12 (with and without metal oxides) was tested to investigate its action mode focused on the fibroblast PS externalization (apoptotic cell death). The flow cytometry results align with [34] and confirm that the viability of cells treated with PA12 remained at 71.16–72.75% (Figure 8) regardless of the concentration of metal oxides in the 24 h extract solution. So, cytometric results (Figure 7 and Figure 8) and colorimetric data (Figure 9) confirmed that PA12 (with and without oxides) is not toxic to fibroblasts [35]. This fact leads to the conclusion that these PA12 composites induce naturally cell apoptosis, counterstain with PI is not visible in Figure 7 and Figure 8. The antifungal concentration of 4 wt % of metal oxides in PA12 induced apoptosis and increased the early and late apoptosis population vs. untreated cells, suggesting its potential effect as a non-toxic antimicrobial agent.

## 4. Conclusions

This paper describes the thermoplastic composites of PA12 mixed with 1–5 wt % of metal oxides (CuO, ZnO, and TiO_2_) fabricated by the melt-blending method. To obtain as high as possible biocidal activity, the used oxides occur in the nanosize, but after the extrusion process they tend to agglomerate. Therefore, SEM images show homogenously dispersed oxides in the PA12 matrix but in the form of the agglomerates. Their diameter decreases with oxides concentration, which is associated with the increased shear stress caused by higher viscosity. This growth in the viscosity of the composites was measured by rotational rheology and was the highest for 5 wt % of the oxides which were characterized by the lowest agglomerates diameter of 4.29 ± 1.69 µm. Due to the presence of the agglomerates, the interactions between polyamide macromolecules and oxides are not sufficient to affect the viscoelastic properties of PA12 since storage and loss modulus remain almost unchanged for the composites. What is more, the loss modulus is larger than the storage modulus and it leads to the conclusion that the composites of PA12 and metal oxides will behave more like viscous rather than elastic liquids. Such a property is positive from applying these polymeric composites in the standard processing methods use for the thermoplastic polymers. What is more, there are only slight changes in the temperatures and crystallinity behavior in the presence of metal oxides. It was confirmed that metal oxides are well-wetted by PA12, and the composites became less hydrophilic in the presence of hydrophobic oxides. Surface studies indicate interaction of the PA12 amide groups with metal cations resulting in the reduction of the copper surface, selective bonding with zinc, and bonding to amide groups for titania. At 4 wt % of the oxides, the contact angle was the lowest (80°). At the same time for this composite, the highest biocidal activity against the representatives of bacteria, fungi, and viruses was reported. As the effect of 4 wt % oxides, the quantitative antimicrobial activity depicts the reduction of the *E. coli* by 87%, *C. albicans* by 53%, and virus *Herpes simplex 1* by 90%. Flow cytometry also proved that cell viability remained at 71.16–72.75% in the presence of PA12 + 4 wt % oxides and that composites are not toxic for the fibroblasts. Studying the reported toxicity of the commonly used silver particles, the mixture of metal oxides seems to be a promising and safe substitute. It needs to be highlighted that PA12 composites demonstrate high biocidal activities and desired physicochemical properties. At the same time, these composites can be processed into the final products such as injected molded parts for the hospitals and nonwovens used as filters or filaments for 3D printing.

To sum up, the main achievements were highlighted below:

- PA12 composites containing CuO, TiO_2_ and ZnO prepared by melt-blending
- Obtaining the biocidal activity against *E. coli*, *C. albicans* and *Herpes simplex 1*
- The lack of toxicity of the composites for the human cells
- Founding the highest activity and the lowest contact angle at 4 wt % oxides
- A slight effect of the oxides on the viscoelastic properties and thermal stability

## Figures and Tables

**Table 2:** Atomic concentrations of the elements at the surface of powder oxides (filler) before and after contact with melted PA12.

State	Atomic Concentration [%]					
	C	O	Cu	Zn	Ti	N
before	8.5	45.7	32.4	10.1	3.3	0.0
after	83.1	10.7	0.4	0.2	0.1	5.6

**Table 3:** The summary of qualitative and quantitative microstructure analysis.

Metal Oxides	SEM Image of Composite Pellets	Histogram of Composite Pellets	Average Grain Size [µm]
1 wt %	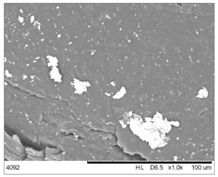	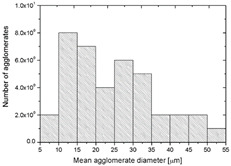	24.8 ± 11.8 pellets 30.9 ± 12.3 after injection molding
2 wt %	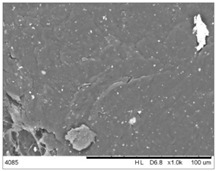	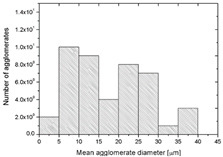	18.2 ± 9.70 pellets 23.8 ± 6.56 after injection molding
3 wt %	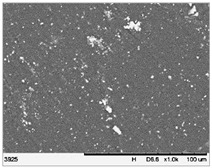	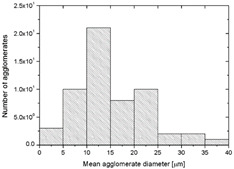	15.4 ± 7.36 pellets 19.2 ± 6.92 after injection molding
4 wt %	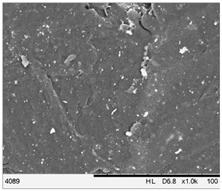	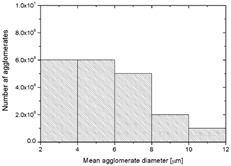	5.56 ± 2.47 pellets 13.9 ± 7.45 after injection molding
5 wt %	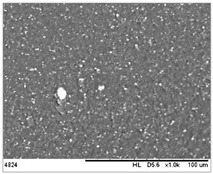	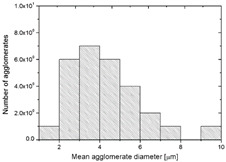	4.29 ± 1.69 pellets 14.6 ± 3.76 after injection molding

**Table 4:** The results of thermal analysis.

	TGA			DSC					
Material	T_5%_ [°C]	T_10%_ [°C]	T_d_ [°C]	1st Heating			2nd Heating		Cooling
				T_g_ [°C]	T_m_ [°C]	X_c_ [%]	T_m_ [°C]	X_c_ [%]	T_c_ [°C]
Pure PA12	406	417	450	48.2	180	32.1	179	33.8	154
PA12 + 1 wt % oxides	406	420	452	43.2	180	26.0	179	27.2	156
PA12 + 2 wt % oxides	403	418	450	42.5	180	29.2	179	29.1	157
PA12 + 3 wt % oxides	402	418	451	46.1	180	26.0	178	27.9	156
PA12 + 4 wt % oxides	404	418	451	43.0	180	27.5	178	27.2	156
PA12 + 5 wt % oxides	399	417	451	47.0	180	27.9	179	28.2	156

## Data Availability

Not applicable.

## Supplementary Materials

The following supporting information can be downloaded at: https://www.mdpi.com/article/10.3390/polym14153025/s1, Figure S1: TGA curve of neat PA12; Figure S2: TGA curve of PA12+1 wt % metal oxides; Figure S3: TGA curve of PA12+2 wt % metal oxides; Figure S4: TGA curve of PA12+3 wt % metal oxides; Figure S5: TGA curve of PA12+4 wt % metal oxides; Figure S6: TGA curve of PA12+5 wt % metal oxides.
