# Supplementary material for: The Composites of Polyamide 12 and Metal Oxides with High Antimicrobial Activity
Source: Polymers (Basel). 2022 Jul 26;14(15):3025. doi: 10.3390/polym14153025 (PMC9330415; doi:10.3390/polym14153025)
Supplement: Supplementary file 1 [file polymers-14-03025-s001.zip › polymers-1751658-supplementary.pdf]

SUPPLEMENTARY DATA

The TGA curves obtained for PA12 composites containing 1-5wt% metal oxides

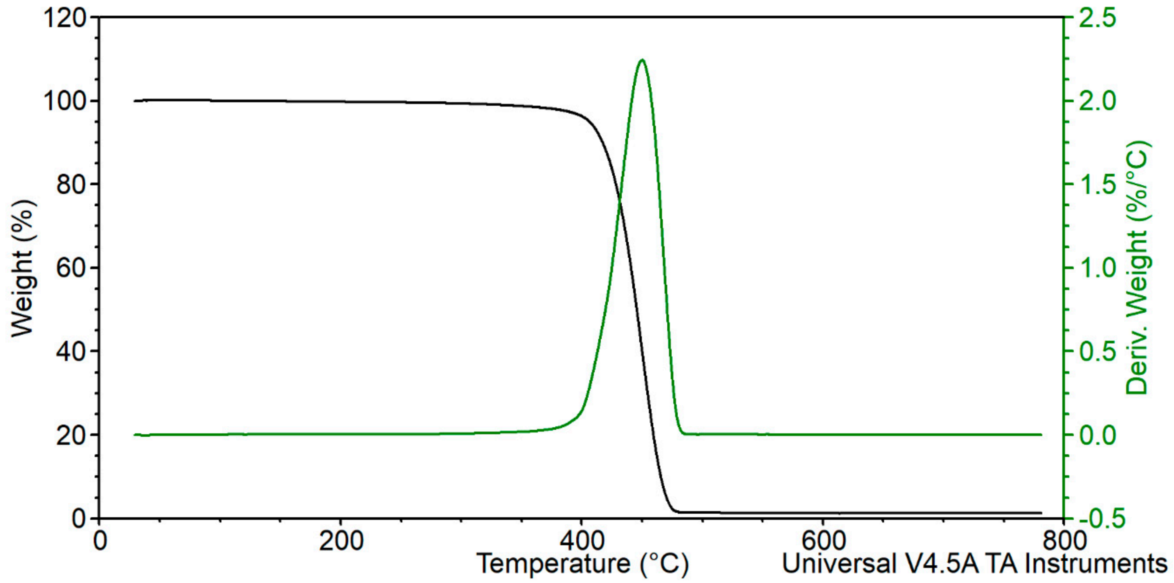

Figure S1: TGA curve of neat PA12.

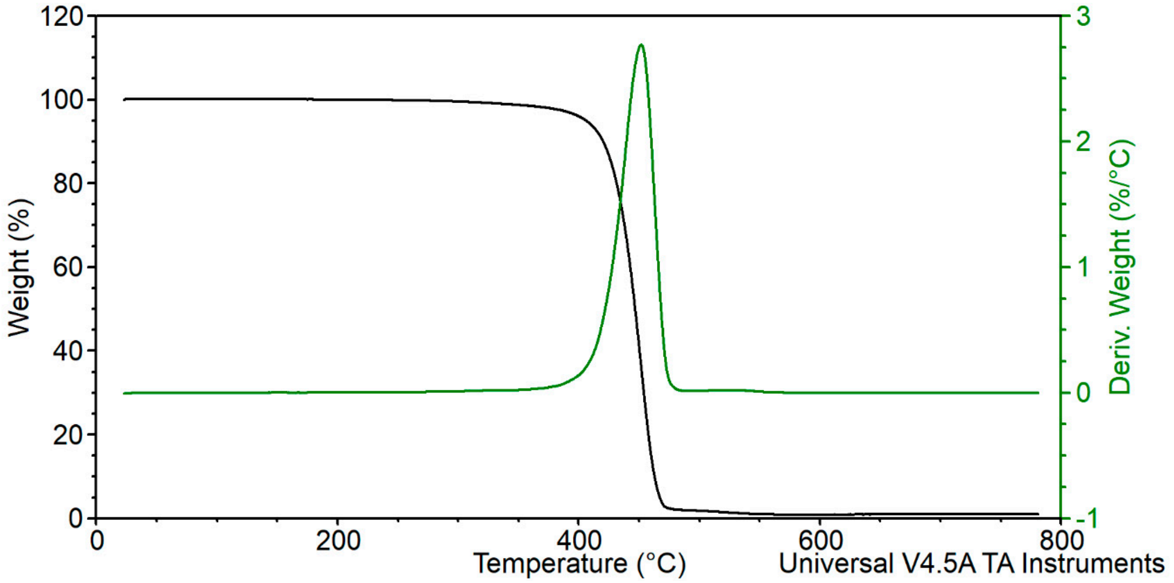

Figure S2: TGA curve of PA12+1wt% metal oxides.

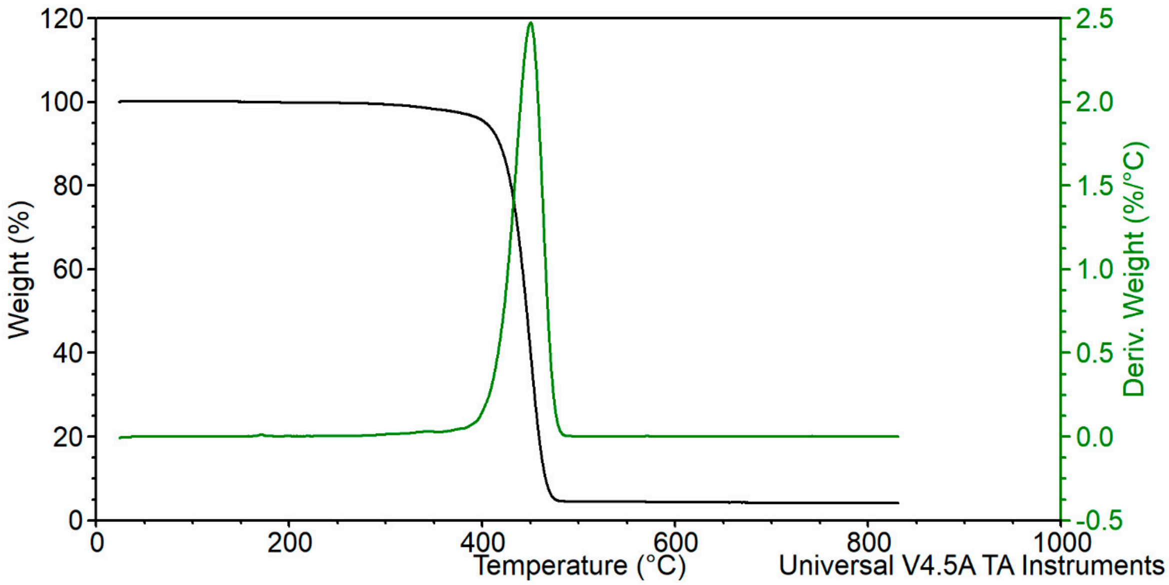

Figure S3: TGA curve of PA12+2wt% metal oxides.

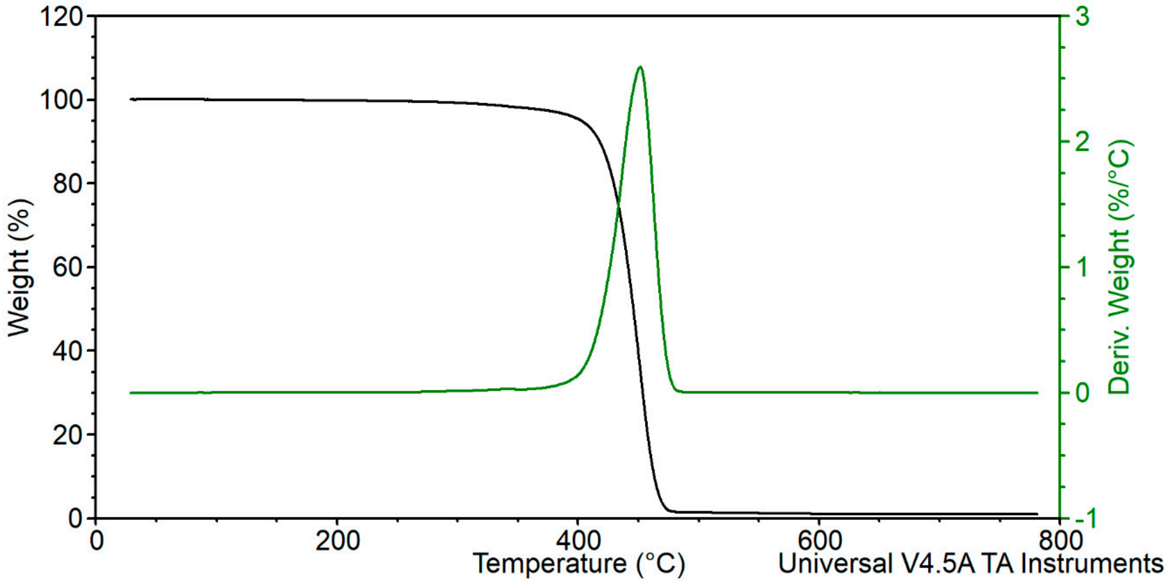

Figure S4: TGA curve of PA12+3wt% metal oxides.

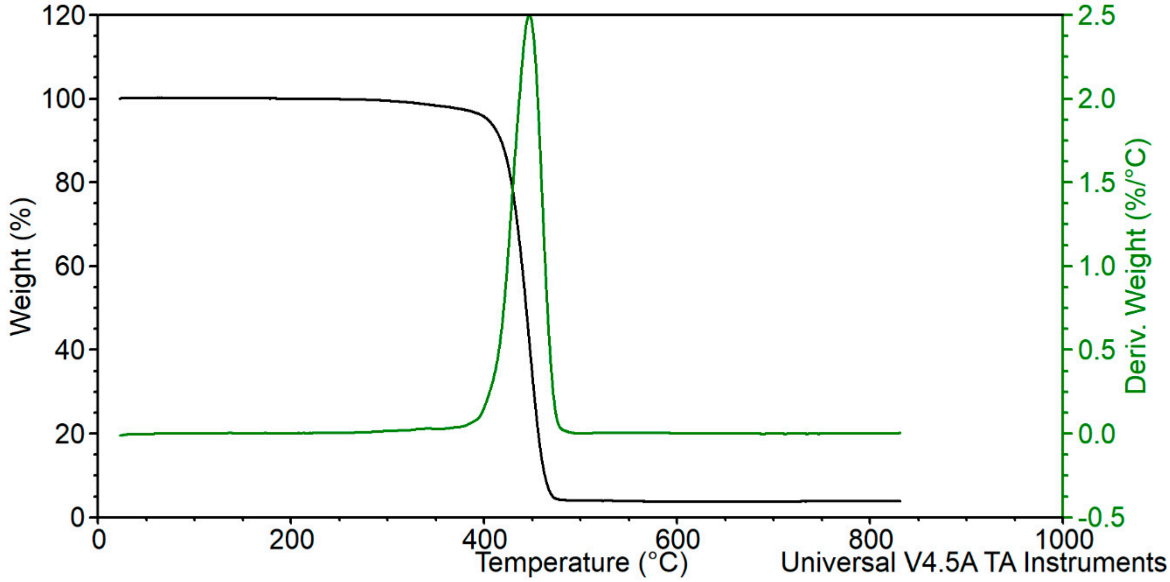

Figure S5: TGA curve of PA12+4wt% metal oxides.

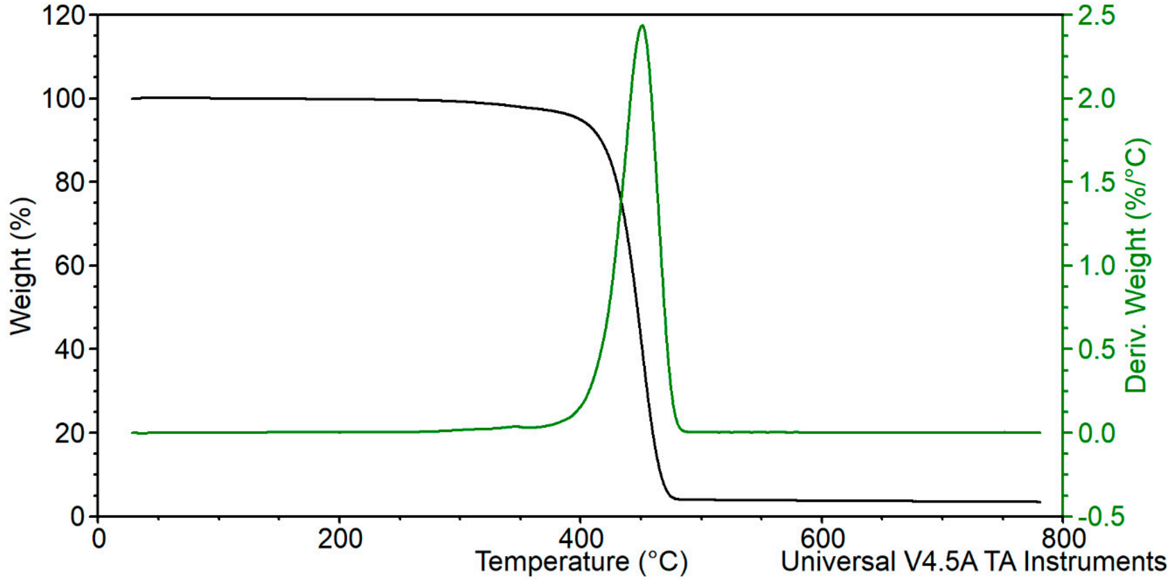

Figure S6: TGA curve of PA12+5wt% metal oxides.
